# Pathophysiology of Bullous Pemphigoid: Role of Type 2 Inflammation and Emerging Treatment Strategies (Narrative Review)

**DOI:** 10.1007/s12325-024-02992-w

**Published:** 2024-10-19

**Authors:** Victoria P. Werth, Dédée F. Murrell, Pascal Joly, Renata Heck, Jamie M. Orengo, Marius Ardeleanu, Verena Hultsch

**Affiliations:** 1 Perelman School of Medicine at the University of Pennsylvania, 3400 Civic Center Blvd, South Pavilion, 1st Floor, Philadelphia, PA 19104 USA; 2 Corporal Michael J. Crescenz Veterans Affairs Medical Center, Philadelphia, PA USA; 3 St George Hospital, University of New South Wales, Sydney, NSW Australia; 4 Rouen University Hospital and INSERM 1234, Normandy University, Rouen, France; 5 Hospital de Clínicas de Porto Alegre, Porto Alegre, RS Brazil; 6 Regeneron Pharmaceuticals Inc., Tarrytown, NY USA; 7 Sanofi, Cambridge, MA USA

**Keywords:** Autoimmune blistering disease, Bullous pemphigoid, Type 2 inflammation

## Abstract

Bullous pemphigoid (BP) is an autoimmune blistering disease that most often affects elderly individuals and has a significant negative impact on quality of life. The disease is characterized primarily by autoantibodies to hemidesmosomal proteins BP180 and/or BP230, and an inflammatory reaction with notable features of type 2 inflammation, including elevated serum IgE, increased numbers of eosinophils in lesions and peripheral blood, and elevated expression of type 2 cytokines and chemokines in skin lesions. In this review, we present what is known about BP pathophysiology, including the role of type 2 inflammation, and discuss how findings from studies of biologics targeting type 2 immune mediators have helped to clarify the biological mechanisms driving BP pathophysiology. Future studies of these targeted therapies and others in development will help to further elucidate the mechanisms underlying BP pathophysiology and potentially provide better treatment options for patients.

## Key Summary Points

**Table:** 

Bullous pemphigoid (BP) is an autoimmune blistering disease that predominantly affects the elderly and severely impacts their quality of life.
This narrative review presents the current knowledge on BP pathophysiology, focusing on the role of type 2 inflammation.
The review shows how research on biologics targeting type 2 immune mediators has shed light on the biological mechanisms driving BP pathophysiology, suggesting new potential treatment options.
Future research on these targeted therapies will be crucial to fully elucidating the mechanisms underlying BP and developing more effective treatments for patients.

## Introduction

Bullous pemphigoid (BP) is an autoimmune blistering disease with elements of type 2 inflammation that mainly affects elderly individuals (generally those over 50 years of age) [1–4]. In most patients with BP, the disease manifests with a prodromal, nonbullous phase characterized by pruritic, eczematous, excoriated, urticaria-like lesions that can progress to a generalized, pruritic, bullous eruption; however, some patients remain in the eczematous and urticarial stage without developing blisters [3]. Skin lesions in BP and associated symptoms, notably pain and pruritus, can have a significant negative impact on patients’ quality of life [5].

BP pathophysiology is not completely understood but is characterized primarily by the production of immunoglobin G (IgG) autoantibodies directed against hemidesmosomal anchoring proteins BP antigen 180 (BP180) and/or BP antigen 230 (BP230) [6, 7]. Several lines of evidence also suggest a prominent type 2 inflammatory response in BP. Type 2 inflammation predominantly involves the activation of group 2 innate lymphoid cells, T helper type 2 cells, eosinophils, and inflammatory cytokines such as interleukin (IL)-4, IL-5, and IL-13 [8]. In patients with BP, immunoglobin E (IgE) and eosinophils were elevated in both peripheral blood and skin lesions, and levels of inflammatory cytokines IL-4, IL-5, and IL-13 were also increased in skin lesions [8–10].

Currently available BP treatments (e.g., anti-inflammatory agents, immunosuppressants) are limited by side effects, including immunosuppression, lack of efficacy for some patients, and relapses (including relapses during treatment as well as relapses following treatment cessation) [11]. Given that BP is most prevalent in elderly populations, treatment strategies for BP should consider the unique health concerns of elderly individuals, including the high burden of comorbidities and associated safety concerns, polypharmacy risks, age-related changes in drug metabolism, and heightened risks associated with immune suppression [12].

## Immune Dysregulation in BP

BP is characterized primarily by IgG autoantibodies to BP180 and/or BP230, which are components of hemidesmosomes involved in dermal-epidermal cohesion [6, 7, 13]. Nearly all patients with BP have circulating IgG autoantibodies to BP180 (especially to the BP180-NC16a extracellular domain), and studies support a pathogenic role of BP180 in BP pathogenesis [11, 14, 15]. Specifically, studies of mouse models of BP demonstrate that mice injected with murine BP180 IgG develop subepidermal blistering that closely resembles that seen in patients with BP, and studies of patients with BP reveal that serum levels of autoantibodies to BP180-NC16a correlate with BP disease activity [14, 15]. IgG subclass 4 (IgG4) is the most predominant subclass of IgG autoantibodies in BP. However, the role of IgG4 in BP pathogenesis is not entirely clear. The pathogenic mechanisms involved in blister formation following BP autoantibody binding are complex and can be subdivided into complement-dependent and complement-independent mechanisms [13]. Since IgG4 antibodies have a limited ability to activate complement mechanisms, it is likely that IgG4 induces blister formation through complement-independent mechanisms [16, 17]. Elevated serum levels of IgE are also seen in patients with BP, with anti-BP180 and anti-BP230 IgE autoantibodies playing an important role [18–20]. Of note, BP disease severity correlates with elevated levels of IgE autoantibodies specific to BP180-NC16a [21, 22]. The role of IgE autoantibodies in BP was validated in a human skin graft–mouse model in which human IgE autoantibodies from BP sera injected into human skin grafted onto mice induced erythema and elevated plaques similar to the clinical features of BP [19]. Recent research also suggests that IgE and BP180 form immune complexes in BP skin, which may activate mast cells and eosinophils through the high-affinity IgE receptor FcεRI [23].

Inflammatory cell infiltration (including mast cells, neutrophils, and eosinophils) is a consistent feature of skin lesions in BP, and studies suggest that these cells play an important role in BP blister formation [24]. The involvement of mast cells in BP pathophysiology is well documented, although their precise role is likely highly complex [25]. Mast cell infiltration and degranulation in BP lesional skin is observed in patients with BP and mice injected with anti-mBP180 antibodies, and mast cell degranulation leads to the release of inflammatory cytokines and proteases that contribute to epithelial barrier damage [25–27]. The use of mast cell stabilizer sodium cromoglycate has reduced pruritus and relapses in some cases of BP, further supporting the role of mast cells in BP pathophysiology [28]. Additionally, mast cells may be involved in recruiting other inflammatory cells into lesional skin; studies show that mast cell activation precedes neutrophil and eosinophil infiltration, and inhibition of mast cell degranulation in mice prevents neutrophil infiltration and subsequent blister formation [27]. Consistent with a prominent role for neutrophil recruitment in BP pathophysiology, studies show that blocking neutrophil infiltration in mice prevents anti-mBP180-induced subepidermal blistering, and intradermal administration of neutrophil chemoattractant IL-8 in mice that are resistant to the pathogenic activity of anti-mBP180 IgG induces subepidermal blistering [29].

In addition to mast cells and neutrophils, eosinophils likely play an important role in BP pathophysiology. Eosinophils are abundant in BP skin lesions and the peripheral blood of patients with BP, and eosinophil numbers correlate with BP disease severity [30]. Eosinophils may amplify local type 2 inflammation in BP skin lesions by releasing cytokines and chemokines (eotaxin and MCP-4) that act in a positive feedback loop by recruiting more eosinophils [31]. Studies show that eosinophils are necessary for anti-BP180 IgE-mediated skin blistering, and that eosinophils participate in dermal–epidermal junction separation through the generation of reactive oxygen species, eosinophilic granule release, and eosinophil extracellular trap formation [32, 33]. Eosinophils are also an important source of IL-31 (an important driver of pruritus), and some studies have found elevated levels of IL-31 in blister fluid and serum in patients with BP [34, 35]; however, Kulczycka-Siennicka et al. reported conflicting results, with lower serum IL-31 levels in patients with BP compared with healthy controls [36]. Eosinophils in BP may also release toxic proteins, such as major basic protein, eosinophil cationic protein, and eosinophil peroxidase, which can activate mast cells directly via the Mas-related G protein-coupled receptor X2 [37–39].

Several lines of evidence point to an important role of type 2 inflammatory cytokines and chemokines in BP pathophysiology [40–45]. In BP lesions, levels of type 2 inflammatory cytokines IL-4, IL-5, and IL-13 are elevated, as are levels of chemokines CCL11 (also called eotaxin 1), CCL26 (eotaxin 3), CCL13 (monocyte chemoattractant protein 4, or MCP-4), and CCL17 (thymus- and activation-regulated chemokine, or TARC) [40–45]. IL-4 and IL-13 are key and central drivers of type 2 inflammation in multiple diseases, and they might be involved in BP as well [46–48]. BP skin lesions show enhanced homing of IL-4- and IL-13-producing T cells [10], and studies show that IL-4 and IL-13 induce the production of eotaxins CCL11, CCL24, and CCL26 in human lung endothelial cells [48]. Consistent with this finding, dual blockade of IL-4 and IL-13 blocks expression of proinflammatory cytokines IL-5, IL-6, and IL-33 and eotaxins CCL11 and CCL24, and prevents eosinophil infiltration into lung tissue in mice [47, 48]. Given that eotaxins strongly attract eosinophils and that IL-5 plays an important role in eosinophil differentiation, maturation, and proliferation, it is thought that these mediators may play an important role in blood and tissue eosinophilia in patients with BP. Indeed, studies show that eotaxin levels in BP blister fluid correlate with the number of dermal infiltrating eosinophils [42]. IL-4 and IL-13 have also been shown to upregulate FcεRI expression on the cell surface of mast cells, and IL-4 enhances cytokine and chemokine production by mast cells [49]. Additionally, sensory neurons can be directly activated by IL-4 and IL-13, and activated neuronal IL-4 receptor subunit α (IL-4Rα) is critically involved in chronic pruritus by sensitizing sensory neurons to other pruritogens [50]. While IL-4 and IL-13 can directly influence human mast cell transcriptomes, independent of IgE crosslinking, each cytokine can also potentiate the effects of IgE crosslinking and alter gene expression. Notably, alone or in combination with IgE crosslinking, IL-4 was more potent at inducing inflammatory gene expression compared with IL-13, suggesting a more dominant role for IL-4 in mast cell activation and priming [47]. IL-4 and IL-13 can also induce immunoglobin isotype switching to IgE and IgG4 in B cells [51, 52].

Patients with BP have a high prevalence of neurological comorbidities [53–55], and studies link levels of anti-BP180 and anti-BP230 IgG with neuropsychiatric and neurological conditions such as Alzheimer’s disease, Parkinson’s disease, and stroke [56–59]. Despite the well-established role of neuroimmune interactions in other atopic diseases (such as atopic dermatitis [AD], prurigo nodularis [PN], asthma, chronic rhinosinusitis with nasal polyps [CRSwNP], and eosinophilic esophagitis [EoE]) [60], it is still unclear if neuroimmune interactions can lead to BP pathogenesis.

## Current Treatment Modalities

The 2022 European guidelines’ recommended treatments for BP include high-potency topical corticosteroids whenever possible, and orally administered prednisone as an alternative. Immunosuppressive therapies (including methotrexate, azathioprine, mycophenolate mofetil, or mycophenolate acid) may be used in case of contraindications or resistance to corticosteroids. The treatment arsenal also includes doxycycline and dapsone (though their use is controversial), B cell-depleting therapy, and intravenously administered immunoglobulin (IVIG), and, more recently, biologics are being explored as treatment options for patients with BP [61].

## Targeted Therapies for BP Treatment

**Fig. 1:**
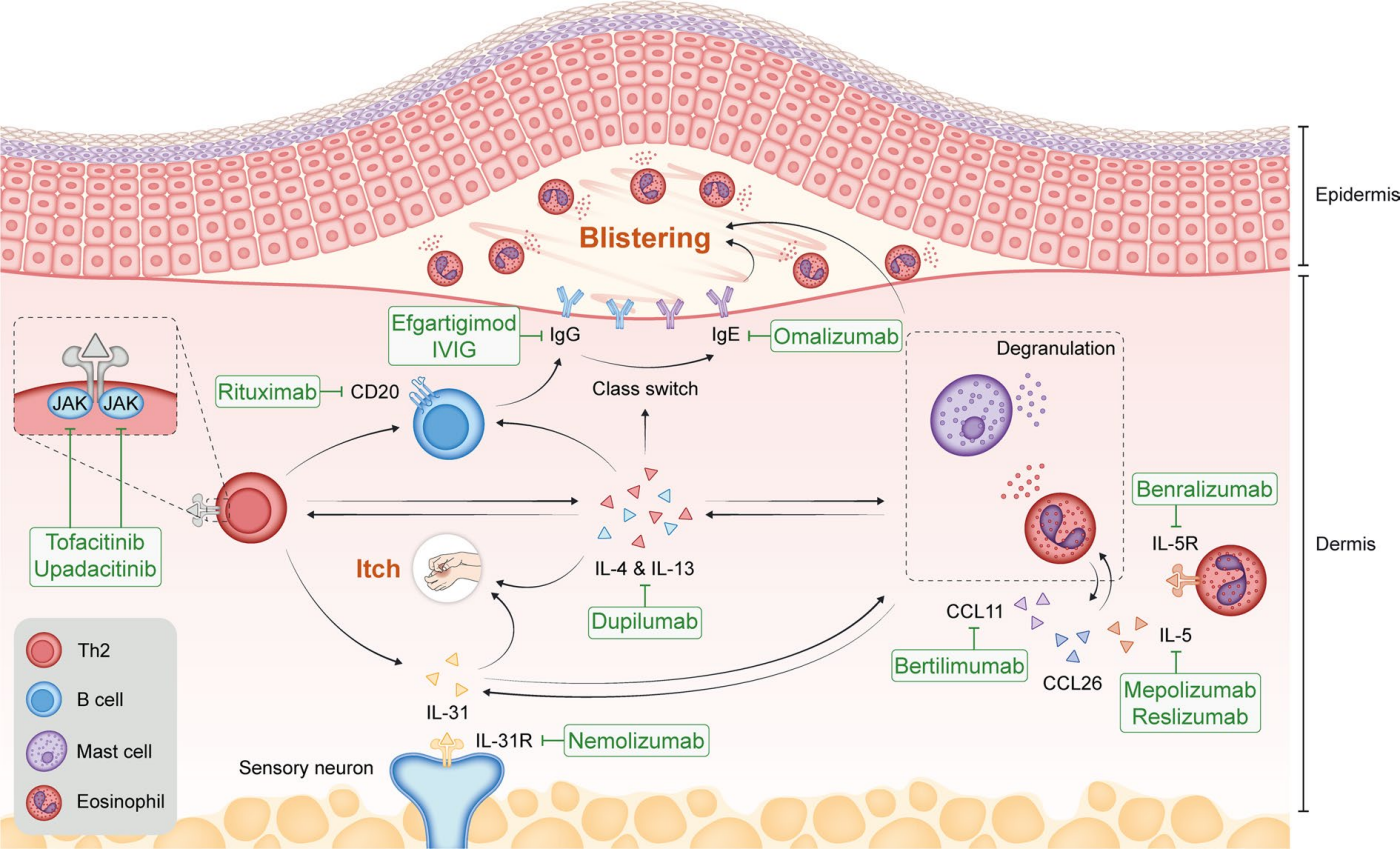
Some examples of targeted therapies that are currently under investigation or have been investigated for the treatment of BP. *Rituximab* targets the CD20 receptor on B lymphocytes, inducing B cell depletion and preventing differentiation into plasma cells. *Dupilumab* inhibits IL-4 and IL-13 signaling, downregulates eosinophil chemotaxins, Th2-associated chemokine activity, B cell proliferation, and autoantibody production, and improves pruritus by reducing IL-13 and IL-31. *Omalizumab* binds to IgE, reducing total IgE levels and eosinophilia. *Nemolizumab*, an anti-IL-31 receptor A antibody, reduces pruritus. *Reslizumab* and *mepolizumab* target IL-5, affecting eosinophil maturation, activation, and chemotaxis. *Benralizumab* targets IL-5Rα, causing eosinophil and basophil apoptosis. *Bertilimumab* targets CCL11, reducing eosinophil recruitment. *Tofacitinib* and *upadacitinib* inhibit the JAK-STAT pathway, impacting immune cell function and cytokine production. *IVIG* and *efgartigimod* reduce pathogenic IgG. *BP* bullous pemphigoid, *CCL* chemokine (C–C motif) ligand, *CD* cluster of differentiation, *IgE* immunoglobulin E, *IgG* immunoglobulin G, *IL* interleukin, *IL-5Rα* interleukin-5 receptor alpha, *IVIG* intravenous immunoglobulin, *JAK-STAT* Janus kinase-signal transducer and activator of transcription, *Th2* T helper type 2. This figure was adapted from Karakioulaki et al. 2024 [104] under a Creative Commons Attribution-NonCommercial 4.0 International License

Studies of biologics targeting various components of type 2 immunity in the treatment of BP have provided insights into BP pathophysiology and paved the way for better treatment options for patients. These biologics include omalizumab (targets IgE), rituximab (targets cluster of differentiation [CD]20, which is expressed on B cells), bertilimumab (targets CCL11/eotaxin 1), mepolizumab and reslizumab (target IL-5), benralizumab (targets the IL-5 receptor), nemolizumab (targets the IL-31 receptor), and dupilumab (targets IL-4 and IL-13) (Fig. 1).

### Omalizumab

Omalizumab is a monoclonal antibody that binds to IgE and prevents IgE binding to FcεR1, which has been used as an off-label treatment option in some patients with BP. The first case report of a patient with BP successfully treated with omalizumab (subcutaneous injection of 300 mg every 2 weeks [q2w] for 16 weeks) revealed a reduction in body surface area involvement with urticarial plaques, resolution of tense blisters (with some small 4–6 mm erosions remaining), reduction in eosinophils, and no change in IgG by week 16 [62]. Four months after treatment discontinuation, clinical symptoms (including pruritus and blisters) returned, but resolved again once omalizumab treatment was reinstituted. No safety data were reported in the study [62]. A subsequent case series evaluating omalizumab treatment in six patients (with subcutaneous doses ranging from 300 to 375 mg and dosing frequency ranging from q2w to every 8 weeks) found that five of six patients benefited from omalizumab, with a reduction in use of other immunosuppressants, inhibition of new blisters, reduction in pruritus, and reduction in eosinophil counts [63]. One patient experienced epigastric pain and a mild elevation of liver enzymes (aspartate aminotransferase—41, alanine aminotransferase—63), which resolved with treatment [63]. Another case series evaluating omalizumab treatment in 11 patients (subcutaneous 300 mg q2w in 10 of 11 patients, and 375 mg every 4 weeks [q4w] in 1 patient) found complete clearance of skin lesions in 6 of 11 patients (54.5%) and a partial response in 3 of 11 patients (27.3%) following a median duration of 4.4 months of omalizumab treatment [64]. In the same study, omalizumab also reduced percent body surface area involvement and systemic steroid use (all patients reduced their steroid dose, while 50% discontinued steroid use completely). Omalizumab was well tolerated, and 81.8% (*n* = 9/11) of patients did not experience adverse events. Omalizumab was discontinued in one patient who experienced possible omalizumab-induced BP flares, and one patient died as a result of infection while being treated with omalizumab and prednisone [64].

### Rituximab

Rituximab is a monoclonal antibody that binds to cell surface protein CD20, which is widely expressed on B cells. Rituximab binding to CD20 has multiple downstream effects, but the ultimate result is the depletion of B cells. A study of rituximab treatment (1000 mg on days 0 and 14, with 12 months of follow-up) in seven patients with BP found a cessation of new skin lesions and a reduction of steroid use in all patients at 6 months, with no serious adverse events; two patients experienced disease flares at weeks 7 and 11.5 [65]. In a recent retrospective study, 17 patients with refractory BP showed faster disease clearance when receiving rituximab combined with omalizumab compared with rituximab monotherapy. In this study, four patients died in the rituximab monotherapy group (infectious pneumonia [*n* = 2], aspiration pneumonia [*n* = 1], and exacerbation of the underlying disease [*n* = 1]), while none died in the combination therapy group, and remaining patients had no serious adverse events [66]. Another study examining the efficacy of rituximab, as well as the resulting immunological profile of 17 patients with relapsing BP, found complete remission in nine patients 2 years following one cycle of rituximab treatment (of the remaining patients, three had withdrawn from the study and five had died [all deaths occurred in the first year of the trial and were caused by the following: general status alteration, *n* = 2; acute respiratory failure, *n* = 1; cardiac failure, *n* = 1; gastrointestinal bleeding, *n* = 1; and there were two cases of pneumonia that occurred at day 10 and day 270]), a dramatic reduction of B cells lasting 9 to 12 months post treatment, and a decrease in IL-6, IL-15, and tumor necrosis factor alpha-expressing, BP180-specific B cells alongside the appearance of IL-10 and IL-1RA-expressing, BP180-specific, immunoglobulin M-positive B cells in patients in complete remission [67]. A study of off-label rituximab treatment (intravenous 375 mg/m^2^ weekly [qw]) in two patients with BP—a 2-year-old and a 63-year-old—demonstrated efficacy, with complete clearance of lesions in the child following 13 months of treatment [68]. Adverse events observed in the child during rituximab treatment included noninfective secretory enteropathy requiring parenteral nutrition, parainfluenzal pneumonia, varicella-zoster virus sepsis with pulmonary and meningeal involvement, and hypogammaglobulinemia. In the adult patient, blister formation stopped, and erosions started to heal by 4 weeks of rituximab treatment; however, the patient developed *Clostridium difficile* enteropathy and died 2 weeks later as a result of hospital-acquired bacterial pneumonia [68]. Finally, a study of rituximab treatment (intravenous 375 mg/m^2^ qw for 4 weeks) in two patients with BP found complete clearance of lesions in both patients and no serious side effects [69]. These findings suggest that rituximab is highly effective at depleting B cells in patients with BP and that BP180-specific B cells undergo functional changes following rituximab treatment.

### Bertilimumab

Bertilimumab is a monoclonal antibody targeting CCL11 (eotaxin 1). As described above, CCL11 is involved in the recruitment of eosinophils to skin lesions in BP. In a single-group, phase 2 clinical trial of bertilimumab in patients with moderate-to-severe BP (*n* = 9), patients were treated with bertilimumab (intravenous 10 mg/kg, on days 0, 14, and 28, with 13 weeks of follow-up). At day 84, 86% of subjects demonstrated at least a 50% improvement in bullous pemphigoid disease area index (BPDAI) activity score and 57% showed at least a 90% improvement [70]. Bertilimumab also improved pruritus and quality of life, was well tolerated (only one serious adverse event occurred and was not considered related to bertilimumab), and all patients were able to reduce their steroid use. On the basis of these results, bertilimumab was granted fast-track designation by the US Food and Drug Administration for the treatment of BP. These results support the role of eosinophil recruitment to skin lesions in BP pathogenesis. However, the development of bertilimumab for the treatment of BP by Immune Pharmaceuticals was halted as a result of company financial concerns.

### Dupilumab

Dupilumab is a fully human VelocImmune®-derived [71, 72] monoclonal antibody that binds IL-4Rα, the shared receptor component for IL-4 and IL-13, and prevents signaling of both IL-4 and IL-13 [47]. Dupilumab clinical trial data have shown that IL-4 and IL-13 are key drivers of type 2 inflammation, which plays a major role in AD, asthma, CRSwNP, EoE, and PN [73, 74]. In a retrospective cohort study (*n* = 146 patients), 52 (35.6%) patients achieved complete remission following treatment with dupilumab (300 mg q2w following an initial dose of 600 mg), with 9% experiencing relapse during the observation period. Within this study, 27% of patients reported adverse events, with infections and eosinophilia being the most frequently noted [75]. In patients with BP, two individual case studies showed successful treatment with dupilumab (subcutaneous 300 mg qw in one case and 300 mg q2w in the other case), although safety data were not reported in either study [76, 77]. In a small case series of dupilumab treatment (*N* = 13), patients were treated subcutaneously with dupilumab 300 mg q2w (which was increased to 300 mg qw in some partial responders) [78]. Total disease clearance was observed in 7 of 13 patients (53.8%), and 92.3% (*n* = 12/13) achieved disease clearance or a satisfactory response; specific monitoring for adverse events was not performed, and adverse events were not reported or documented in patients’ medical records [78]. An additional retrospective case series (*n* = 24 patients) of dupilumab (subcutaneous 300 mg q2w) plus methylprednisolone (0.6 mg/kg/day) and azathioprine (2 mg/kg/day) or methylprednisolone and azathioprine alone found that combination therapy with dupilumab was statistically superior to methylprednisolone and azathioprine alone. Although an adverse event of osteoporosis was recorded in the dupilumab group, no adverse events related to dupilumab were reported in the study [79]. Finally, another case study in a patient with BP revealed complete disease clearance with combination therapy of omalizumab (subcutaneous 300 mg q4w) and dupilumab (subcutaneous 300 mg q2w); no safety data were reported [80]. Taken together, monotherapy efficacy results from studies of dupilumab thus far support the role of IL-4 and IL-13 in BP pathogenesis.

### Benralizumab

Benralizumab is a monoclonal antibody that binds to IL-5 receptor subunit α, which is expressed on eosinophils and basophils. Studies have shown that benralizumab induces apoptosis through antibody-dependent, cell-mediated cytotoxicity, thereby reducing the levels of circulating eosinophils and basophils [81, 82]. Benralizumab is approved by the US Food and Drug Administration for the add-on maintenance treatment of patients with severe eosinophilic asthma [83]. In studies of patients with eosinophilic asthma, benralizumab reduced rates of asthma exacerbations and depleted eosinophils [82]. Phase 3 trials of benralizumab for the treatment of BP began in 2021 (NCT04612790) but were terminated because of lack of efficacy. Furthermore, on the basis of a case study of a patient who developed BP while being treated with benralizumab for bronchial asthma [84], paradoxical development of BP could be a risk during benralizumab treatment.

### Mepolizumab and Reslizumab

Mepolizumab and reslizumab are monoclonal antibodies that bind to IL-5, thereby blocking IL-5 signaling. A phase 2, 16-week trial investigated the efficacy and safety of mepolizumab (intravenous 750 mg q4w) as an add-on therapy to oral corticosteroids in patients with an acute BP flare-up (*n* = 32 patients; NCT01705795) [85]. No difference was found between mepolizumab and placebo in the time to relapse, the primary endpoint, or the cumulative rates of patients achieving disease control and maintaining disease control. Treatment with mepolizumab significantly reduced blood eosinophil numbers without affecting tissue eosinophil infiltration [85]. Adverse events were reported in all patients in both treatment groups, which was likely due to the age of the patient population, and regarding events associated with mepolizumab, 13 events were possible, one was likely, and one was certain [85]. Another patient with BP treated with a single dose of reslizumab (intravenous 3.5 mg/kg) showed a rapid improvement in skin lesions, suggesting that IL-5 blockade may be efficacious in some patients with BP. No safety data were reported in this case study [86].

### Nemolizumab

Nemolizumab, a monoclonal antibody against IL-31 receptor A, is approved in Japan for the treatment of AD-associated pruritus and is under clinical development for the treatment of AD and PN [87]. Some studies have found increased levels of IL-31, released primarily from eosinophils, in blister fluid and lesional skin from patients with BP. IL-31 may affect eosinophil function [88, 89]. However, other reports have also found reduced levels of IL-31 in BP compared with healthy volunteers [36]. Another case report found that a patient with prurigo-like AD and asthma developed BP after receiving nemolizumab treatment. Administration of dupilumab in this case to treat BP resulted in continued remission [90]. Given these findings, nemolizumab could be a potential therapy for some patients with pruritus in BP in the future; however, further studies are required to examine this possibility.

### JAK Inhibitors

Janus kinase (JAK) inhibitors are a group of small molecules that inhibit the JAK-signal transducer and activator for the transcription pathway, which contributes to immune cell functioning, including cytokine biosynthesis [91]. In one study, seven patients with recalcitrant BP showed alleviation of pruritus after receiving treatment with JAK inhibitor tofacitinib, and a decrease in levels of autoantibodies and eosinophils with a sustained complete remission over a 14-month follow-up period. No severe adverse events were observed in this study [92]. In a case report, a patient with recalcitrant BP showed a response to JAK inhibitor upadacitinib in the form of resolving urticoid eruption, healing erosions, and no active blisters or mucosal progression at 4 weeks. Additionally, this case report did not provide safety data [93]. Finally, another case report in a patient with BP treated with upadacitinib (while tapering off prednisone over 20 days) showed a complete resolution of disease at the 2-month follow-up visit, with no new blister formation since upadacitinib initiation and no remaining itch. After 5 months of upadacitinib treatment, the skin continued to heal, and the patient had not experienced disease recurrence or flares. No adverse events were noted in this patient [94]. Additional controlled clinical trials are needed to investigate the efficacy and safety of JAK inhibitors in patients with moderate-to-severe BP.

### Additional BP Therapies

In addition to therapies targeting type 2 inflammation, there are several other treatments for BP, including IVIG therapy and FcRn inhibitors such as efgartigimod (a human IgG1-derived antibody Fc-fragment targeting the Fc receptor that reduces IgG) [95, 96].

### IVIG

IVIG is thought to be effective in relapsing BP by reducing the effect of pathogenic IgG [97–99]. A randomized, placebo-controlled, double-blind trial of IVIG in patients with BP (*n* = 56) found that the disease activity score on day 15 (DAS15) significantly improved for IVIG versus placebo, including when analyzing only severe cases. Adverse drug reactions occurred in 37.9% (*n* = 11/29) versus 18.5% (*n* = 5/27) of patients for IVIG versus placebo, and the most common adverse drug reactions occurring in 5% or more of patients in either treatment arm were liver disorder (10.3% vs 3.7%), decreased platelet count (10.3% vs 0.0%), abnormal hepatic function (6.9% vs 0.0%), fever (6.9% vs 0.0%), and increased blood lactate dehydrogenase (6.9% vs 0.0%) [100]. The most common adverse events with IVIG reported in other studies include mild reactions such as pyrogenic reactions, and minor systemic reactions such as headache, myalgia, fever, chills, low back pain, nausea and/or vomiting, and changes in blood pressure and tachycardia [101, 102]. Several rare, severe, and potentially fatal events have been reported with IVIG, including anaphylactic reactions, thrombotic events, and renal failure [101, 102].

### FcRn Inhibitors

FcRn inhibitors reduce the recycling of IgG in various cells, thereby rapidly reducing total IgG. FcRn inhibitors such as efgartigimod (a human IgG1-derived antibody Fc-fragment targeting the Fc receptor that reduces IgG) resulted in a reduction of serum IgG in healthy volunteers in a phase 1 study and showed promising results in a phase 2 trial in pemphigus [96, 103]. In the phase 1 study, a single administration of efgartigimod reduced IgG levels up to 50% from baseline, and multiple injections resulted in a decrease of up to 75% from baseline. The most common adverse events observed in more than one patient were headache, chills, dizziness, fatigue, abnormal differential white blood cell count, and increased C-reactive protein levels [96]. In the phase 2 study including patients with pemphigus, efgartigimod treatment as monotherapy or in combination with prednisone reduced serum total IgG and anti-desmoglein autoantibodies and led to disease control in 90% (*n* = 28/31) of patients after a median of 17 days, and prolonged treatment in combination with prednisone resulted in complete remission in 64% of patients (*n* = 14/22) within 2–41 weeks [103]. Adverse events were reported in 84% and 87% of patients treated with efgartigimod 10 mg kg^−1^ and 25 mg kg^−1^, respectively, and were mostly mild in severity [103]. Phase 2/3 clinical trials of efgartigimod (NCT05267600) are currently ongoing in adult patients with moderate-to-severe BP, but results are not yet available.

## Concluding Remarks

The development of biologics for the treatment of BP is ongoing, but results from studies thus far have helped elucidate the pathophysiology of BP and support the role of the involvement of type 2 inflammation. The efficacy of omalizumab suggests that IgE plays a role in BP pathophysiology, but the partial or nonresponse in some patients suggests that other mechanisms likely also contribute and may highlight the heterogeneous nature of the disease. Findings from studies of rituximab also support a role for B cells. The efficacy of CCL11 blockade with bertilimumab suggests that eosinophil trafficking to lesions is likely an important component of BP pathophysiology, but the lack of large, controlled studies makes it difficult to fully understand the relative contribution of eosinophil trafficking in BP. IL-5 blockade with both mepolizumab and reslizumab reduced peripheral eosinophil counts in patients with BP, though mixed results suggest that IL-5 blockade may be efficacious in only some patients with BP and that circulating eosinophils may not play a major role in BP pathophysiology. This possibility is further supported by the lack of efficacy of benralizumab, which is thought to work primarily by depleting circulating eosinophils and basophils. Data from the use of JAK inhibitors and IVIG highlight the importance of autoantibodies and eosinophils, and dual inhibition of IL-4 and IL-13 with dupilumab supports the role of these cytokines in BP pathophysiology. Inspired by the IVIG mechanism of preventing IgG autoantibody recycling, the FcRn inhibitor efgartigimod is currently being assessed in patients with BP. Future studies of these biologics and others will help to elucidate the mechanisms underlying BP pathophysiology and provide better treatment options for patients. The use of some of these treatments may be limited by their association with certain adverse events and/or serious adverse events. More randomized controlled studies are needed to further elucidate the efficacy and safety of most of these treatments when used in patients with BP. The information included in this article is based on previously conducted studies and does not contain any new studies with human participants or animals performed by any of the authors.

## Data Availability

Data sharing is not applicable to this article, as no datasets were generated or analyzed during the study.
